# Sodium Dodecyl Sulfate (SDS)-Loaded Nanoporous Polymer as Anti-Biofilm Surface Coating Material

**DOI:** 10.3390/ijms14023050

**Published:** 2013-02-01

**Authors:** Li Li, Soeren Molin, Liang Yang, Sokol Ndoni

**Affiliations:** 1 Danish Polymer Centre, Department of Chemical and Biochemical Engineering, Technical University of Denmark, Kgs. Lyngby DK-2800, Denmark; E-Mail: li.li@nanotech.dtu.dk; 2 Department of Systems Biology, Technical University of Denmark, Kgs. Lyngby DK-2800, Denmark; E-Mail: sm@bio.dtu.dk; 3 Singapore Centre on Environmental Life Sciences Engineering (SCELSE), Nanyang Technological University, Singapore 637551, Singapore; 4 School of Biological Sciences, Nanyang Technological University, Singapore 639798, Singapore; 5 Department of Micro and Nanotechnology, Technical University of Denmark, Kgs. Lyngby DK-2800, Denmark

**Keywords:** nanoporous polymer, SDS, biofilm, *Escherichia coli*

## Abstract

Biofilms cause extensive damage to industrial settings. Thus, it is important to improve the existing techniques and develop new strategies to prevent bacterial biofilm formation. In the present study, we have prepared nanoporous polymer films from a self-assembled 1,2-polybutadiene-*b*-polydimethylsiloxane (1,2-PB-*b*-PDMS) block copolymer via chemical cross-linking of the 1,2-PB block followed by quantitative removal of the PDMS block. Sodium dodecyl sulfate (SDS) was loaded into the nanoporous 1,2-PB from aqueous solution. The SDS-loaded nanoporous polymer films were shown to block bacterial attachment in short-term (3 h) and significantly reduce biofilm formation in long-term (1 week) by gram-negative bacterium *Escherichia coli*. Tuning the thickness or surface morphology of the nanoporous polymer films allowed to extent the anti-biofilm capability.

## 1. Introduction

Most pathogens in the natural environment reside in surface-attached communities known as biofilms [1]. Biofilms can protect pathogens from disinfection and allow microorganisms injured by environmental stress and disinfectants to recover and grow [2]. Once biofilms are formed, it is almost impossible to eradicate them. Biofilms cause extensive damage to industrial settings and place a huge burden on our society [3].

Microbial attachment to a surface is a universal phenomenon in nature and is essential for biofilm formation. In the past years, a series of coating methods have been developed to reduce microbial attachment [4–6]. For example, poly (ethylene oxide) brushes [7], quaternary ammonium salt (QAS) moieties [8] and hydrophilic polyurethanes [9] are reported to reduce microbial attachment as passive coatings. However, the effectiveness of passive coatings for reducing microbial attachment varies greatly since their physicochemical properties can be masked by an adsorbed conditioning film.

Recently, coatings that actively release antimicrobial agents are widely recognized as alternative approach to reduce microbial attachment [10]. Various active coating strategies have been developed with different complexity and these active coatings can release fluxes of antimicrobial agents for long periods (weeks to months) [11–13]. However, certain limitations exist in these active coatings due to their chemical and physical properties. For example, some of these active coatings have low delivery efficiency since a large part of the drug is not able to diffuse through the pores of the polymer [11]. Also, the preparation of many of these active coatings is quite specific and is only suitable for a certain class of antimicrobial agents.

The aim of this work is to develop a universal and simple anti-biofilm coating strategy, providing a potentially better alternative for the existing surface coating systems. The principle is to use nanoporous polymers that are derived from self-organized block copolymers, which can be repeatedly loaded with the desired anti-biofilm agents, as surface coating materials. Block copolymers, due to the chemical incompatibility of the covalently bonded blocks, can self-assemble into well-defined structures, e.g., spheres, cylinders, lamellae, *etc.*, with controllable sizes in the range of 5–100 nm. The nanoporous matrices can be created by totally or partially removing one of the blocks with different methods, as reviewed by Hillmyer [14]. With some attractive features, e.g., large interior surface area, tunable pore size, narrow pore size distribution, and adjustable chemical and mechanical properties, this kind of nanoporous polymers is being extensively explored for a wide range of applications, for instance, templates for nanostructured hybrid materials [15], substrates for catalysis [16], filtration membranes for sustainable water treatment [17], and use in medical diagnostics [18].

In the present work, we explored the potential as anti-biofilm materials of nanoporous polymers templated from block copolymers and loaded with a common anti-biofilm detergent, sodium dodecyl sulfate (SDS). Nanoporous films of different thickness and surface morphology were first loaded with SDS from aqueous solutions. We examined then how efficiently the SDS release from the different nanoporous films in fact prevents the formation of *Escherichia coli* and *Staphylococcus epidermidis* biofilms. SDS as an anti-biofilm agent is directly relevant for industrial applications. Of course, for medical applications other agents should be used instead of SDS and in that context the present serves as a model system.

## 2. Results and Discussion

### 2.1. Preparation of Nanoporous Polymer

**Figure 1:**
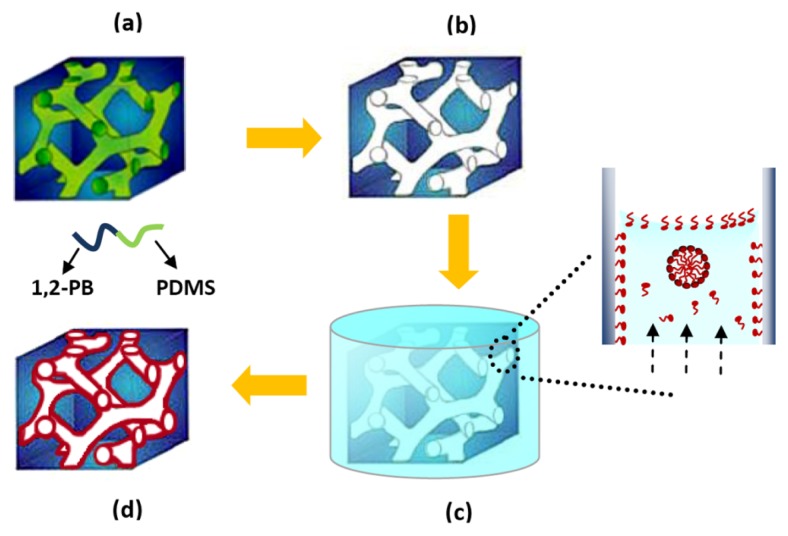
Schematic illustration of the fabrication of nanoporous 1,2-PB polymer and Sodium dodecyl sulfate (SDS) loading process by diffusion-controlled mechanism: (**a**) the precursor 1,2-polybutadiene-*b*-polydimethylsiloxane (1,2-PB-*b*-PDMS) copolymer self-assembles into gyroid morphology at the cross-linking temperature; (**b**) a nanoporous matrix template from the cross-linked copolymer by selectively and quantitatively removal of PDMS; (**c**) nanoporous 1,2-PB matrix in contact with SDS aqueous solution and the loading process shown in the enlarged window; (**d**) SDS-loaded nanoporous 1,2-PB matrix; the red color indicates a SDS adsorption layer.

As depicted in Figure 1, a gyroid nanoporous matrix was prepared from self-assembled 1,2-polybutadiene-*b*-polydimethylsiloxane (1,2-PB-*b*-PDMS) via quantitative and selective removal of the PDMS block (Figure 1a,b). A gyroid nanostructure was designed from the polymer synthesis stage [19] in order to ensure isotropic percolation with no need for structure alignment. The composition of the nanoporous matrix is essentially hydrocarbonic and is therefore hydrophobic, showing a static contact angle of 119° with water [20]. Therefore water cannot spontaneously wet and fill the nanoporous volume, while methanol can [19,20]. For this reason SDS loading was realized by first conditioning the nanoporous 1,2-PB film in methanol before dipping it into SDS aqueous solution (Figure 1c). We have recently reported a detailed study on the load-release of SDS into-from nanoporous cross-linked 1,2-PB [21]. At the loading conditions applied in the present study SDS is adsorbed onto the hydrophobic pore walls creating a dense monolayer as depicted in the inset of Figure 1c. At equilibrium more than 99% of the SDS inside the pores is in the adsorbed state, the rest being either free molecules or organized in micelles [21]. After SDS loading the nanoporous film (Figure 1d) was then challenged in the culture medium with the biofilm model organism *E. coli* to evaluate its efficiency on preventing bacterial attachment and biofilm formation. As already mentioned virtually all SDS inside the nanopores is adsorbed on the pore walls; its release in the presence of water is slow and happens by multiple release-adsorption steps [21]. More information on the SDS release kinetics will be given in the Experimental Section.

**Figure 2:**
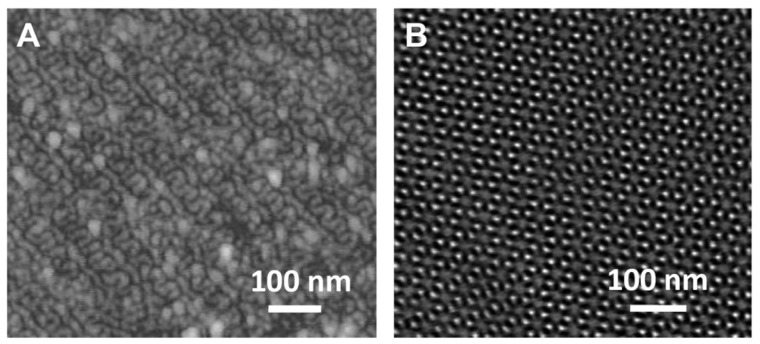
(**A**) Atomic Force Microscopy (AFM) image of film’s cross-section showing the “knitting” projection of gyroid morphology; (**B**) Transmission Electron Microscopy (TEM) micrograph of an ultrathin section of a nanoprous film showing the “wagon-wheel” projection of gyroid morphology.

Typical AFM and TEM images of nanoporous 1,2-PB films are presented in Figure 2, showing a gyroid nanoporosity in the matrix. Two projections of gyroid morphology, the so-called knitting view [22] (Figure 2A) and wagon-wheel view [22] (Figure 2B) were observed. Both images show regular patterns with uniform pore size of ~10 nm. The films used in the present work were identical in the bulk morphology, porosity, pore size and size distribution.

### 2.2. Effect of SDS on Inhibiting Biofilm Formation by *E. coli* Sar18

**Figure 3:**
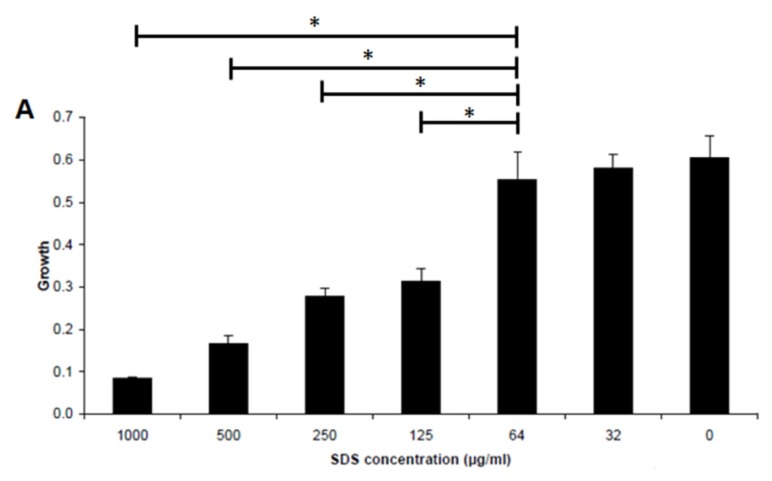

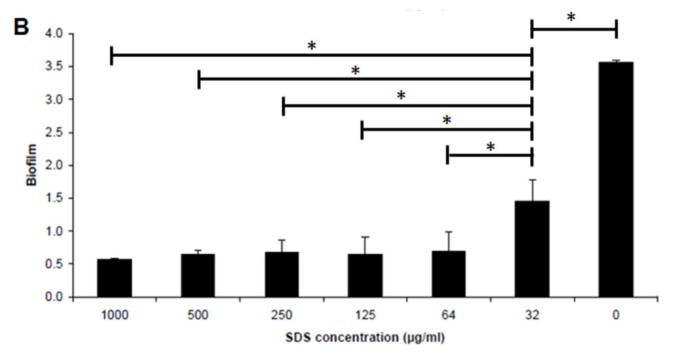
Minimum inhibitoryconcentration (MIC) (**A**) and minimum biofilm inhibitory concentration (MBIC) (**B**) of SDS to *E. coli* Sar18. Bacterium was cultivated in 96 well microtitre tray in AB minimal medium supplemented with 5 g/L glucose at 37 °C for 24 h before measurement. Data are the means and SDs from six replicate wells. * *p* < 0.01.

SDS is an anionic surfactant used in many cleaning and hygiene products and is shown by several studies to inhibit bacterial biofilm formation and disperse mature biofilms [23–25]. We firstly grew the biofilm model organism *E. coli* Sar18 in microtitre tray in medium supplemented with different concentrations of SDS. In our experimental conditions the minimum inhibitory concentration (MIC) and minimum biofilm inhibitory concentration (MBIC) of SDS to *E. coli* Sar18 were 1000 μg/mL and 64 μg/mL, respectively (Figure 3). SDS at subinhibitory concentration (32 μg/mL) could significantly inhibit biofilm formation by *E. coli* Sar18 (Figure 3B).

### 2.3. Attachment Inhibition by SDS-Loaded Nanoporous Film

**Figure 4:**
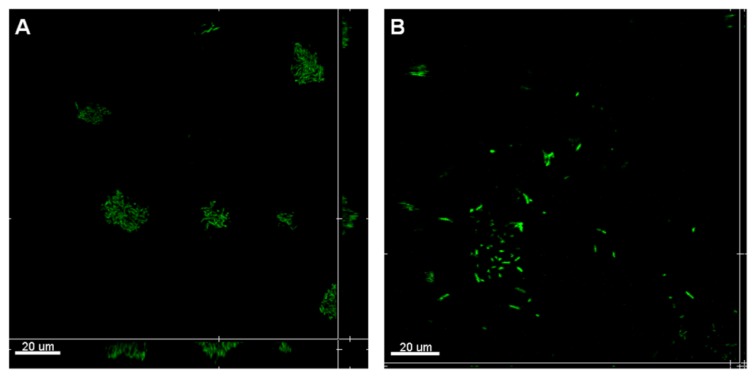
Three-hour attachment assay by *E. coli* Sar18 on T_1_ nanoporous films without (**A**) and with (**B**) loaded SDS. The central pictures show horizontal optical sections, and the flanking pictures show vertical optical sections. Bars, 20 μm.

Shortly after 3 h, *E. coli* Sar18 was able to attach to the surface of control sample T_1_ and start to form microcolony structures (Figure 4A). The microcolony formation is often the essential step for biofilm maturation, which can further lead to formation of thick and resistant biofilms [26,27]. In contrast, the SDS-loaded T_1_ sample could significantly inhibit the formation of microcolony structures by *E. coli* Sar18 (Figure 4B), reducing biofilm development and maturation.

The results of the attachment assay (Figure 4) clearly showed the capabilities of SDS detergent in inhibiting the growth of *E. coli* Sar18 cells, preventing the formation of microcolony structures as well as detaching the cells from the surface. SDS can disrupt bacterial cell-to-cell communications through pili and nanotubes, which are required for aggregation and biofilm formation [28–30]. As a detergent, SDS might also extract or induce denaturation of bacterial surface proteins required for attachment and biofilm formation. SDS is widely used in industrial cleaners and household detergents and thus SDS-loaded nanoporous films might be a potential anti-fouling coating material.

### 2.4. Biofilm Inhibition by SDS-Loaded Nanoporous Film

To evaluate the impact of release of SDS-loaded nanoporous film on biofilm formation*,* we tested SDS-loaded nanoporous films of different thickness (T_1_ 0.5 mm, T_2_ 1.0 mm and T_3_ 1.5 mm). Besides, two samples with thickness of 1.0 mm (referred to as S_skin_) were glued in the opposite way relative to the other samples, *i.e.*, with the skin-layer surface on the upper side, thus exposing only the dense surface to the culture medium.

**Figure 5:**
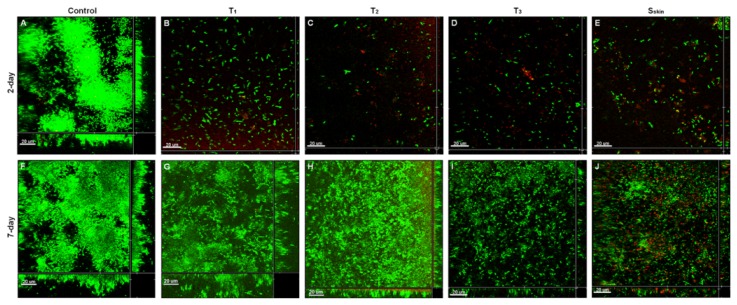
Two-day (**A**–**E**) and 7-day (**F**–**J**) biofilm formation by *E. coli* Sar18 on nanoporous films with (**B**–**E**, **G**–**J**) and without (**A**, **F**) loaded SDS. Green and red cells indicate live and dead cells respectively. The central pictures in each frame show horizontal optical sections, and the flanking pictures show vertical optical sections. Bars, 20 μm. **A**–**E** are freshly prepared control T_1_ nanoporous film, SDS-loaded T_1_ nanoporous film, SDS-loaded T_2_ nanoporous film, SDS-loaded T_3_ nanoporous film, and SDS-loaded S_skin_ nanoporous film, respectively. **F**–**J** are 7-day samples, control T_1_ nanoporous film, SDS-loaded T1 nanoporous film, SDS-loaded T_2_ nanoporous film, SDS-loaded T_3_ nanoporous film, and SDS-loaded S_skin_ nanoporous film, respectively.

After 2-day cultivation, the control sample showed large continuous *E. coli* Sar18 biofilms with dense ball-shaped microcolonies on the surface (Figure 5A). However, only thin layers of *E. coli* Sar18 cells were formed on the surface of the SDS-loaded samples for all the different thickness (T_1_, T_2_ and T_3_) and the skin surface (S_skin_) as shown in Figure 5B–E. A large portion of these cells were dead, as demonstrated by the red color. These results indicate that all of the tested SDS-loaded nanoporous films were able to release SDS and create surface concentrations high enough to prevent the biofilm formation on the surface after a contact time of 2 days.

**Figure 6:**
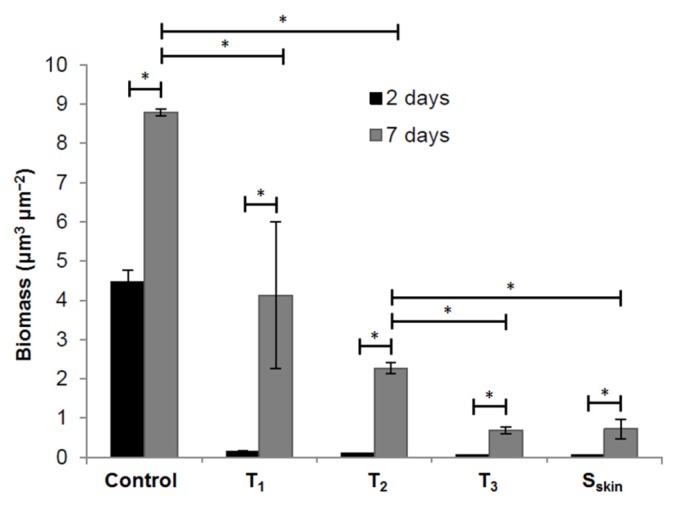
Quantification of biofilms by COMSTAT. The results are means of datasets obtained from analysis of six Confocal Laser scanning Microscope (CLSM) images acquired at random positions in each of the biofilms. Data are the means and SDs from six CLSM images. * *p* < 0.01.

After 7-day release, all the SDS-loaded samples T_1_, T_2_, T_3_ and S_skin_ showed cell attachment and biofilm formation on their surfaces (Figure 5G–J). However, compared to the control sample without the incorporation of SDS (Figure 5F), the SDS-loaded samples were able to reduce the biofilm formation (Figures 5G–J and 6). *E. coli* Sar18 formed least biofilm on the samples T_3_ and S_skin_ (Figures 5I–J and 6). Particularly, a certain amount of dead cells were visible in biofilms formed on the sample S_skin_ (Figure 5J).

The nanoporous matrix plays a positive role in both providing large storage space for SDS molecules due to the large interior surface area and regulating the transport at the molecular level thus offering opportunity of sustainable or even controllable release. The gyroid nanoporosity of the films has the advantage of isotropic percolation which ensures high delivery efficiency. This is definitely supported by the results from the 2-day incubation (Figure 5), which reveal the efficiency of the SDS-releasing nanoporous 1,2-PB films against bacterial attachment and biofilm formation by *E. coli*. Remarkable reduction in bacterial attachment and no visible biofilm formation were found compared with the control sample. It therefore can be expected that the surface concentration of SDS could retain a value within 2 days, which is at least comparable to the MBIC value of 64 μg/mL. This can actually be confirmed by the estimation of the initial SDS surface concentration and the SDS-released concentration after 48 h, as described in “Materials and Methods” section. The estimated value of *C*_os_ (*t* = 0) is almost identical to the MIC value, and 15 times higher than MBIC. The size of a bacterium is usually of the order of few micrometers and at such near-surface zone the concentration can be very close to *C_os_*. Therefore, the expectation from the above estimation is that the starting SDS concentration is sufficient to kill bacteria in the near-surface zone and to totally inhibit biofilm formation. The estimated average SDS concentration after 48 h is close to the MBIC value, and it’s therefore consistent with the absence of biofilm formation in the 48 h experiment.

Unfortunately, the present SDS-releasing nanoporous systems showed weaker ability against the biofilm formation in 7-day anti-biofilm assay (50% reduction, T_1_) compared to 2- day anti-biofilm assay (95% reduction, T_1_, Figure 6). This can be ascribed to low release rate of SDS after 72 h, as already reported in [21]. However, increasing the film thickness or slowing down the release rate by exposing the skin layer side to the solution did reduce long-term biofilm formation (74% reduction for T_2_, 92% reduction for T_3_ and S_skin_, Figure 6). The improvement provided by the S_skin_ samples is particularly interesting. In a comparative study of glucose permeability through 1,2-PB nanoporous membranes [31], we have observed that the presence of skin layer diminishes the effective diffusion coefficient by a factor of 5.7 compared to the porous surface. This effect is correlated to decreased surface porosity due to the compact nature of the skin layer, irregularly interrupted by porous defects [31].

**Figure 7:**
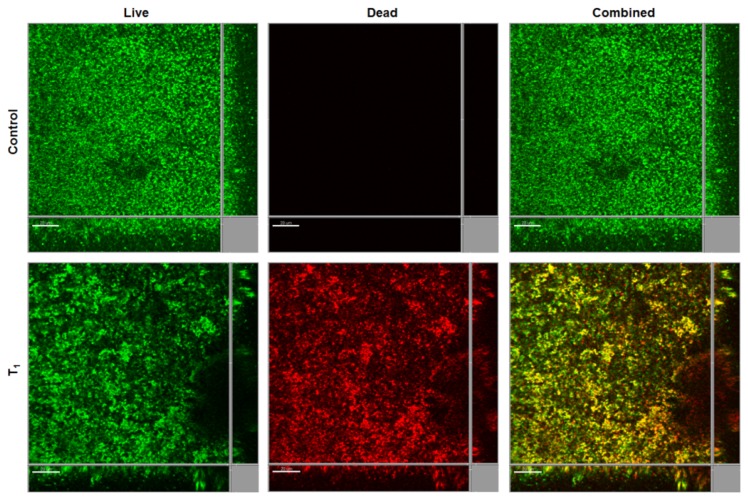
One-day biofilm formation by *S. epidermidis* RP62A on control and SDS-loaded nanoporous T_1_ films. Green and red cells indicate live and dead cells respectively. The central pictures in each frame show horizontal optical sections, and the flanking pictures show vertical optical sections. Bars, 20 μm.

To evaluate the biofilm inhibitory effect of the SDS-loaded nanoporous films on Gram-positive bacteria, we tested the biofilm formation of *Staphylococcus epidermidis* RP62A [32] on the control and SDS-loaded nanoporous T_1_ films. *S. epidermidis* RP62A formed thick biofilms on both control and SDS-loaded nanoporous T_1_ films after 1 day cultivation (Figure 7). However, SDS-loaded nanoporous T_1_ film killed most of the attached *S. epidermidis* RP62A cells (Figure 7). This result indicates that SDS is not a good agent for controlling biofilms formed by *S. epidermidis*. Further test is required to identify agents suitable for preparing anti-*S. epidermidis* nanoporous films.

## 3. Experimental Section

### 3.1. Preparation of Nanoporous Film

The 1,2-polybutadiene-*b*-polydimethylsiloxane (1,2-PB-*b*-PDMS) diblock copolymer was synthesized by living anionic polymerization as previously reported [19]. The general procedure to prepare a nanoporous 1,2-PB matrix was as follows. *A tetrahydrofuran* or oxacyclopentane (THF, Sigma-Aldrich, Copenhagen, Denmark) solution of 1,2-PB-*b*-PDMS and cross-linker (dicumyl peroxide, DCP, Sigma-Aldrich) was solvent casted onto a flat bottom glass Petri-dish. The molar amount of DCP was 1% relative to that of the double bonds in 1,2-PB. After complete evaporation of THF, the cast copolymer film was cross-linked at 140 °C for 2 h under nitrogen atmosphere. The cross-linked copolymer film was subsequently immersed in a tetra-n-butylammonium fluoride solution (TBAF, Sigma-Aldrich) in THF at room temperature for 36 h to selectively and quantitatively remove the PDMS block. The molar amount of TBAF was twice that of Si–O bonds in PDMS. The etched film was rinsed in a mixture of THF and methanol and finally dried under nitrogen flow at room temperature.

As reported previously [31], the outer surface of the resultant nanoporous film showed two different features. A 30 nm dense skin layer was observed on the free outer surface that during cross-linking was in contact with the nitrogen atmosphere; while the surface in contact with the glass petri-dish was nanoporous with a porosity of approximately 40%. In the subsequent biofilm formation assay experiments the tested samples had the porous surface side exposed to the culture medium for releasing SDS, except for one set of samples referred to as S_skin_ that had the surface with the skin layer in contact with the culture medium. In this study, we prepared nanoporous 1,2-PB films with three different thicknesses: 0.5 ± 0.1 mm, 1.0 ± 0.1 mm, and 1.5 ± 0.1 mm. They are denoted in the manuscript as T_1_, T_2_ and T_3_ respectively.

### 3.2. Characterization of Nanoporous Films

The morphology of nanoporous membranes was checked by Atomic Force Microscopy (AFM) and Transmission Electron Microscopy (TEM). AFM was conducted at ambient air using NanoMan AFM in tapping mode, with NANOSENSORS™ SSS-NCH AFM probe. TEM was performed on a FEI TECNAI T20 at an acceleration voltage of 200 kV.

The fracture cross-section of a nanoporous film was trimmed and microtomed to a flat surface for AFM measurement. Ninety nm slices were sectioned from the film and deposited onto a holey carbon copper grid for TEM measurements. The microtoming process was completed on a Leica ultramicrotome with a cryo 35_diamond knife (DIATOME) at room temperature.

### 3.3. Loading of Nanoporous Films with SDS

A systematic study of SDS infiltration from aqueous solutions with SDS concentration between 0.5 and 50 mM into the nanoporous 1,2-PB matrix has been recently reported [21]. It has been shown that the SDS adsorption onto the inner surface of nanoporous 1,2-PB film saturates at concentrations above 6.8 mM. In the present work we used a 10 mM SDS aqueous solution to ensure adsorption saturation. The nanoporous 1,2-PB film was pre-wet with methanol for 10 minutes and then kept under shaking for 15 h in 15 mL of SDS aqueous solution. The SDS-loaded samples were gently wiped with a tissue and dried under nitrogen flow without further treatment.

### 3.4. Minimum Biofilm Inhibitory Concentration (MBIC) Assay

The microtitre tray biofilm formation assay was performed as previously described [33] to measure the minimum biofilm inhibitory concentration of SDS to *E. coli*. Overnight cultures were grown in AB minimal medium [33] supplemented with 5 g/L glucose and diluted to optical density OD 600 nm = 0.001 with fresh medium. SDS was added to culture medium at different concentrations from 0 to 1000 μg/mL. The diluted cultures were transferred to the wells of microtitre trays (150 μL per well) and incubated for 24 h at 37 °C. The growth of bacterium was recorded by measuring absorbance at OD 600 nm for minimum inhibitory concentration (MIC) determination. The medium was then removed from the wells and these were washed twice with 0.9% NaCl, stained with 0.1% crystal violet and again washed twice with 0.9% NaCl; the crystal violet-stained biofilms were then resuspended in 96% ethanol, and biofilm cell-associated dye was measured as absorbance at OD 600 nm by the use of a Wallac microplate reader. Six replicates were used for each SDS concentration.

### 3.5. Attachment Assay

**Figure 8:**
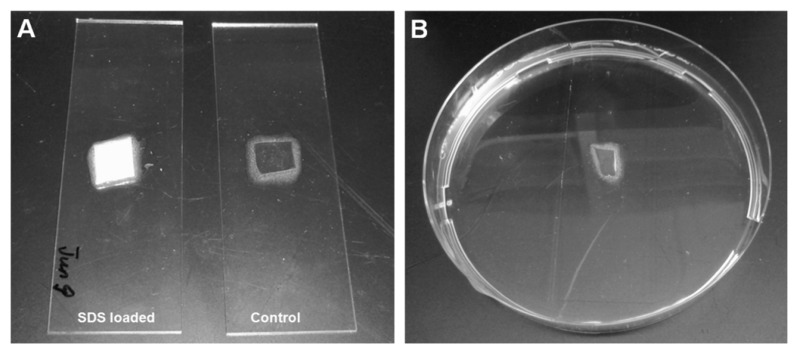
Nanoporous 1,2-PB films with (left) and without (right) the incorporation of SDS glued on the surface of glass slides (**A**) and submerged into bacterial culture in a petri dish (**B**).

Nanoporous 1,2-PB films T_1_ (0.5 ± 0.1 mm) with the incorporated SDS were tested in the attachment assay, while T_1_ samples without SDS were used as control samples. As shown in Figure 8A, the samples were attached onto the surface of glass slides with glue (Epoxy Universal 335, high strength 2-component expoxy adhesive) surrounding the side surfaces. The glue was immediately cured at 65 °C for 2.5 h to allow a complete curing process. As a result, the tested samples were fixed on the glass slides and only the upper surface was exposed for effective release. In this experiment, the upper surface was the porous surface. Prior to be ready for the attachment assay, all the samples were sterilized under UV exposure for 30 min. The biofilm model organism gram-negative *Escherichia coli* Sar18 [34] was used in this study. The samples were submerged into bacterial cultures in Petri dishes as illustrated in Figure 8B. *E. coli* was cultivated in 25 mL AB minimal medium supplemented with 5 g/L glucose at 37 °C for 3 h. After that the slides were taken out from Petri dishes and washed with fresh AB minimal medium in order to remove floating bacterial cells. Three 1,2-PB films were used for each analysis and two confocal images were taken at different positions of each 1,2-PB film.

### 3.6. Biofilm Formation Assay

In the biofilm formation assay we selected different thicknesses of the SDS-loaded nanoporous films for the 2-day and 7-day tests, T_1_ (0.5 ± 0.1 mm), T_2_ (1.0 ± 0.1 mm) and T_3_ (1.5 ± 0.1 mm). The T_2_ and T_3_ films have a higher capacity for SDS than T_1_ enabling longer SDS release periods (data not shown). Film pieces not containing SDS were taken as control samples. All the samples exposed the porous surface to the culture medium for effective release. For comparison we tested two samples S_skin_ (1.0 ± 0.1 mm thick) with the skin-layer side exposed to the *E. coli* culture. For the 2-day assay, nanoporous 1,2-PB films T_1_, T_2_ and T_3_ loaded with SDS as well as a control T_1_ without SDS were glued on the surface of glass slides and submerged into bacterial cultures in Petri dishes as described above. *E. coli* was cultivated in AB minimal medium supplemented with 5 g/L glucose at 37 °C for 2 days. After that the slides were taken out from petri dishes and washed with fresh AB minimal medium in order to remove floating bacterial cells. Three 1,2-PB films were used for each analysis and two confocal images were taken at different positions of each 1,2-PB film. For the 7-day test, we glued the SDS-loaded samples T_1_, T_2_, T_3_ and S_skin_ on glass as above and left them in fresh AB minimal medium for 5 days (SDS-containing medium was replaced by fresh medium every 2 days) and then submerged into bacterial cultures in Petri dishes for additional 2 days. After that the slides were taken out from the Petri dishes and again washed by fresh AB minimal medium in order to get rid of floating bacterial cells. Three 1,2-PB films were used for each analysis and two confocal images were taken at different positions of each 1,2-PB film.

For comparison, biofilm formation by Gram-positive bacterium *Staphylococcus epidermidis* RP62A on the control and SDS-loaded nanoporous T_1_ films was also investigated as described above.

### 3.7. CLSM Observation

The LIVE/DEAD^®^ Bacterial Viability Kit was used to stain live (appears green under fluorescence microscopy) and dead (appears red under fluorescence microscopy) bacterial cells on the surface of all of tested the nanoporous 1,2-PB films. The nanoporous 1,2-PB films were observed under a Carl Zeiss LSM510 META Confocal Laser scanning Microscope (CLSM, company, Jena, Germany) for biofilms. Images were obtained using a 63×/1.4 objective. Simulated three-dimensional images and sections were generated using the IMARIS software package (Bitplane AG: Zurich, Switzerland, 2010).

### 3.8. COMSTAT Analysis

CLSM images were analyzed by use of the computer program COMSTAT for calculating the biomass of biofilms [35]. A fixed threshold value and connected volume filtration were used for all image stacks. Six images were used for analysis of each sample.

### 3.9. Estimation of the SDS Concentration Near the Surface

We estimated the SDS concentration at the outer surface of the nanoporous samples from data reported in a recent publication [21] on the kinetics and equilibrium of SDS load—release in samples similar to the T_1_ samples of this study. Then we compared the estimated values with the minimum inhibitoryconcentration, MIC and with the minimum biofilm inhibitory concentration, MBIC. The equilibrium amount of SDS adsorbed into nanoporous 1,2-PB films immersed in excess SDS aqueous solutions was 23% of the 1,2-PB matrix mass, independent of SDS concentration in the concentration range of 6–50 mM. The adsorbed amount corresponds to a monolayer of SDS onto the pore walls. At the beginning of a release experiment, the SDS concentration at the very outer surface *C*_os_ (*t* = 0) of a nanoporous film fully loaded with SDS can be estimated from the concentration of SDS in the porous volume at the immediate vicinity of the outer surface *C*_is_ (*t* = 0), reduced by a factor equal to the surface porosity α.

(1) $$C_{os}(t=0)=\alpha \cdot C_{is}(t=0)\approx \alpha \cdot CMC=0.4\cdot 8.1\;\text{mM}=3.24\;\text{mM}=934\;\mu \text{g}/{\text{cm}}^{3}$$

The approximation is justified by the already mentioned concentration independence of equilibrium adsorption in a wide range of concentrations, with the lowest value being just below the critical micelle concentration (CMC) of SDS in water (CMC = 8.1 mM). The surface porosity α for the porous outer surface was 0.4 [21]. We used the molar mass of SDS, *M_SDS_* = 288.4 g/mol in order to convert the molar concentration into a mass concentration at the end of the above expression. In the time interval 0 ≤ *t* ≤ 72 h (= 259,200 s) the experimental data on the kinetics of SDS release in excess of distilled water could be accurately described by a power low expression:

(2) $$M(t)=M_{0}[1-0.0114\cdot t{\left(s\right)}^{1/3}]$$

where *M* (*t*)/*M*_0_ is the mass fraction of SDS remaining inside the nanopores at release time *t*. About 70% of the SDS adsorbed could be released after 72 h [21]. The initial mass of adsorbed SDS was 23% of the 1,2-PB matrix, which in the present case was *m*_PB_ = 20 mg, therefore *M*_0_ = 4.6 mg.

The presented SDS release data were obtained under shaking and in the presence of large excess of water. The release experiments of the present study were done in Petri dishes containing 50 mL aqueous solution with stirring. We illustrate the use of the above relation by considering the 48 h release experiment. At the end of the experiment a total of 2.9 mg of SDS was released in the surrounding solution and the corresponding average concentration was 58 μg/mL. The estimated values of initial *C*_s_ and the SDS concentration at different times of release will be compared with the experimental values of MIC and MBIC in the ‘Discussion’ section. The consumption of released SDS by the *E. coli* bacteria was not taken into account in the course of the presented estimates.

## 4. Conclusions

In the present work, we explored the potential as anti-biofilm materials of nanoporous polymers templated from block copolymers and loaded with a common anti-biofilm detergent, sodium dodecyl sulfate (SDS). The presented results are promising and constitute a basis for the development of a simple and generic strategy for the fabrication of anti-biofilm coatings. Supplementary research efforts are necessary in order to further improve the release profile from the nanoporous polymers, thus making our system competitive in practical applications. Guided by the promising results from the skin layer surface we can try to further reduce the pore size of the nanoporous polymer, or to better control the morphology of the skin layer and its defects, thus enabling to decrease the release rate.
